# Exploration of interaction scoring criteria in the CANDO platform

**DOI:** 10.1186/s13104-019-4356-3

**Published:** 2019-06-07

**Authors:** Zackary Falls, William Mangione, James Schuler, Ram Samudrala

**Affiliations:** Department of Biomedical Informatics, Jacobs School of Medicine and Biomedical Sciences, University at Buffalo, 77 Goodell St., Suite 540, Buffalo, NY 14203 USA

**Keywords:** Drug repurposing, Drug-protein interaction, Binding site similarity, Protein structure docking, Molecular fingerprinting

## Abstract

**Objective:**

Ascertain the optimal interaction scoring criteria for the Computational Analysis of Novel Drug Opportunities (CANDO) platform for shotgun drug repurposing to improve benchmarking performance, thereby enabling more accurate prediction of novel therapeutic drug-indication pairs.

**Results:**

We have investigated and enhanced the interaction scoring criteria in the bioinformatic docking protocol in the newest version of our platform (v1.5), with the best performing interaction scoring criterion yielding increased benchmarking accuracies from 11.7% in v1 to 12.8% in v1.5 at the top10 cutoff (the most stringent one) and correspondingly from 24.9 to 31.2% at the top100 cutoff.

**Electronic supplementary material:**

The online version of this article (10.1186/s13104-019-4356-3) contains supplementary material, which is available to authorized users.

## Introduction

Drug discovery is an arduous process that requires many years of effort and costs billions of dollars before new ones are approved for patient use [1, 2]. Recent data indicate that the average cost and time to market for a new drug are about $3 billion and 14 years, respectively [3, 4]. New paradigms are therefore imperative to make drug discovery more efficient and financially sustainable.

As of 2013, there were $\approx$ 1453 human use drugs FDA approved for a variety of indications/diseases with an accompanying trove of data on their safety profiles and efficacy [5]. A vast majority of these drugs are small molecules, which are inherently promiscuous in their potential interactions with macromolecules in their environment, resulting in undesirable off-target or side effects [6–11]. The multitargeting nature of small molecules, and the presence of these off-target effects, provides support for the repurposing of drugs for indications for which they are not approved [7, 11–15]. The cost, time, and, most importantly, risk to go from “bench to bedside” for such repurposed drugs are significantly decreased.

The first version (v1) of the Computational Analysis of Novel Drug Opportunities (CANDO) platform for multitarget shotgun drug repurposing [7, 11, 14–19] implemented a modelling pipeline to predict interactions between 46,784 protein structures and 3733 human use compounds. Various protocols, representing software components, are implemented within each pipeline to calculate an interaction score for each drug-protein pair corresponding to the potential binding affinty. Applying this across entire proteomes results in compound-proteome interaction signatures that are then compared and ranked according to similarity. We then generate new indication associations for drugs based on the similarity of their interaction signatures to drugs with a known indication, i.e., make predictions about putative repurposable therapeutics for every indication with at least one known drug. Furthermore, we quantify the expected accuracy of our predictions by performing a leave-one-out benchmarking procedure which determines whether an associated drug for each known drug-indication pair is captured within a cutoff of a list of compounds sorted by proteomic signature similarity to the “left out” drug.

In the v1 platform, we used an interaction scoring protocol that integrated bioinformatics and cheminformatics tools to calculate $\approx$ one billion scores. We updated our platform to v1.5 by exploring the use of different bio- and cheminformatics software to vary these interaction scores to discover the best performing scoring protocol. The pipelines implementing these new scoring protocols were subsequently benchmarked, the results of which are reported here.

All of the pipelines with the new interaction scoring protocols in CANDO v1.5 yield promising benchmark performance. However, there is some variance depending on how many top putative drug candidates are generated and benchmarked: At the lowest cutoff (top10 putative drug candidates), the pipeline with the best performance uses only the cheminformatics interaction score. At higher cutoffs (top25–top100), the pipeline with the best performance combines the bioinformatic and cheminformatics outputs for the interaction scores. These results help guide future experimental validation studies of the platform by enabling us to select the appropriate interaction scoring protocol based on the number of putative drug candidates to be tested.

## Main text

### Methods

**Fig. 1:**
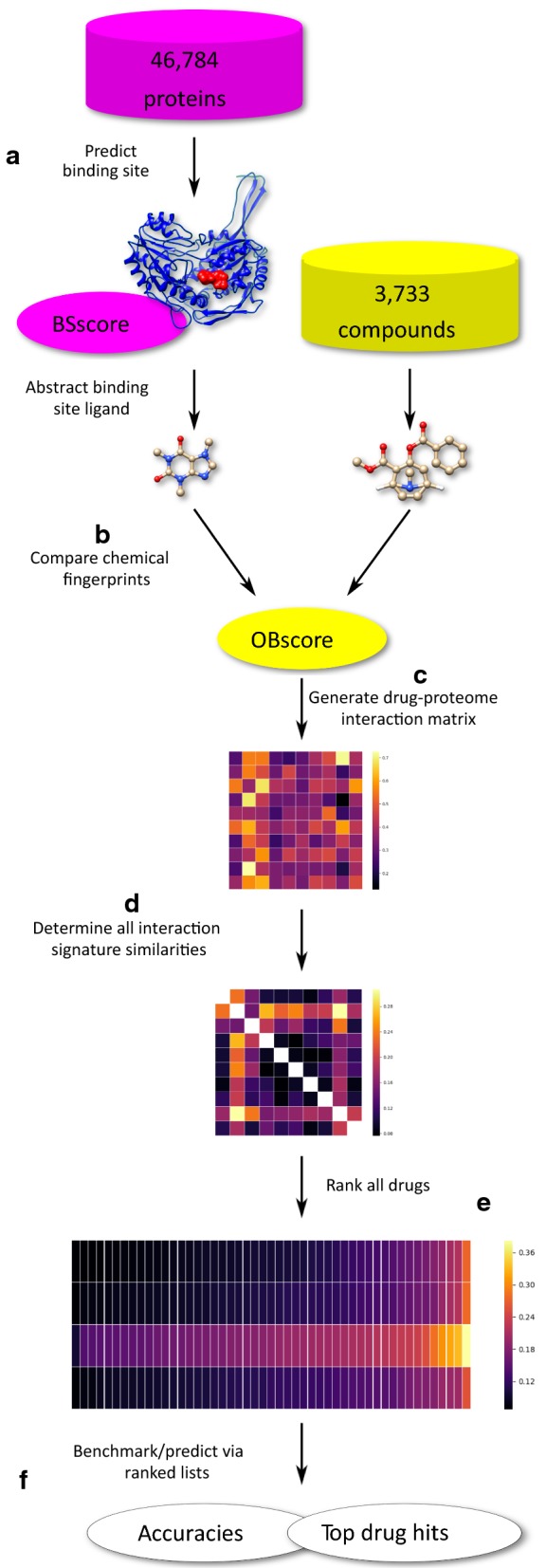
CANDO platform workflow. **a** Binding sites are predicted for each of the 46,784 proteins in the CANDO protein structure library using the bioinformatic tool COFACTOR [20–23], resulting in a BSscore. **b** The native ligand in the predicted binding site is compared to all 3,733 compounds in the CANDO putative drug library by calculating the chemical fingerprints using the FP4 fingerprinting method in Open Babel for each structure, resulting in an OBscore [24]. **c** Each compound-protein interaction is given a score based upon the OBscore and/or BSscore, which is then used to populate the interaction matrix. **d** The similarity score between every pair of compound-proteome interaction signatures (the vectors of 46,784 interaction scores) is calculated by root-mean-squared deviation (RMSD) which are then used to populate the compound-compound similarity matrix. **e** The compound-compound similarities are sorted and ranked by RMSD. **f** Benchmarking is accomplished by measuring the recovery rate of the known approved drugs, *i.e.*, per indication accuracies are obtained based on whether or not pairs of drugs associated with the same indication can be captured within a certain cutoff of each of their ranked compound similarity lists; other similar compounds that fall within a particular cutoff are hypothesized to be repurposeable drugs and serve as predictions. The CANDO platform utilizes a proteomic approach for drug repurposing, with the hypothesis that drugs with similar interaction signatures will behave similarly

The CANDO v1.5 pipeline is outlined and detailed in Fig. 1. Refer to Additional file 1 for more details regarding the CANDO platform and the v1.5 pipeline.

### Ranking drug lists and benchmarking metrics

The RMSDs in each row of the compound-compound similarity matrix (Fig. 1d) are sorted to yield ranked similarity lists for each compound (Fig. 1e). Each drug associated with an indication is left out and checked to see if it is captured within a certain cutoff in the ranked list to any of the other remaining ones [associated with that indication] (Fig. 1f). The cutoffs used typically are top10, top25, top50, and top100, reflecting the top ranked 10–100 similar compounds for a given drug.

This procedure is repeated iteratively for all drugs associated with every indication for a particular cutoff, resulting in the indication accuracy. Mathematically, indication accuracy is calculated using the formula $\frac{c}{d}\cdot 100$, where *c* is the number of times at least one drug with the same indication was captured within a particular cutoff and *d* is the total number of drugs approved for that indication. Taking the mean of these accuracies (for all 1439 indications with at least two approved drugs) gives the average indication accuracy for a pipeline at a particular cutoff.

The other benchmarking metrics used are the average pairwise accuracy which is a weighted average of all indication accuracies based upon the number of approved drugs for each indication, and indication coverage, which is the number of indications with a non-zero accuracy, i.e., at least one approved drug that was left out was successfully recaptured within a cutoff.

#### Differences between versions 1 and v1.5 of the CANDO platform

For v1.5, we use Open Babel for the chemical fingerprint comparison between all compounds and predicted binding site ligands for each protein, compared to using OpenEye ROCS in v1 [16]. Pipeline modifications have been made to leverage OBscore and/or BSscore to populate the interaction matrix in multiple pipelines for CANDO v1.5, whereas only the BSscore was used in CANDO v1 to calculate compound-protein interactions.

The following pipelines were generated in CANDO v1.5: Best OB, Best BS, Best OB+BS, and Best OBxBS. The values in the matrix for each compound-protein interaction in the first two pipelines use the OBscore; Best OB is the highest OBscore between the compound and all predicted binding site ligands for each protein, while Best BS is the OBscore that corresponds to the best local binding site prediction using COFACTOR. The last two pipelines involve adding and multiplying the OBscore and BSscore for each compound-protein interaction; the highest sum or product between the compound and the predicted binding site ligands was chosen as the interaction score.

By removing the cutoffs for interaction scores (BSscore and ROCSscore in CANDO v1 [16]), we decreased the number of compound-protein interactions with zero scores, which we empirically determined had a negative effect on benchmarking performance. Additional minor modifications have been made in CANDO v1.5 software to reduce the number of compounds with all-zero proteome interaction signatures.

### Results

We generated compound-proteome interaction matrices using the BSscore and OBscore interaction scoring schemes to implement the following pipelines: Best OB, Best BS, Best OB+BS, and Best OBxBS. These pipelines were compared to the one used in CANDO v1, as well as random controls, with respect to benchmarking performance using three evaluation metrics: average indication accuracy, average pairwise accuracy, and indication coverage.

#### Comparison of v1 and Best OB pipelines

The CANDO v1.5 Best OB pipeline average indication accuracy is higher at all cutoffs when comparing to the pipeline from CANDO v1, increasing from 11.7 to 12.8% for the top10 cutoff. The relative increase in average indication accuracy for the remaining cutoffs are 3.0% (top25), 4.1% (top50), and 6.3% (top100). The indication coverage for Best OB is greater than v1 at all cutoffs (30–70 more non-zero indications) except top10, where the coverage for v1 and Best OB is about the same at 562 and 563 indications, respectively.

**Fig. 2:**
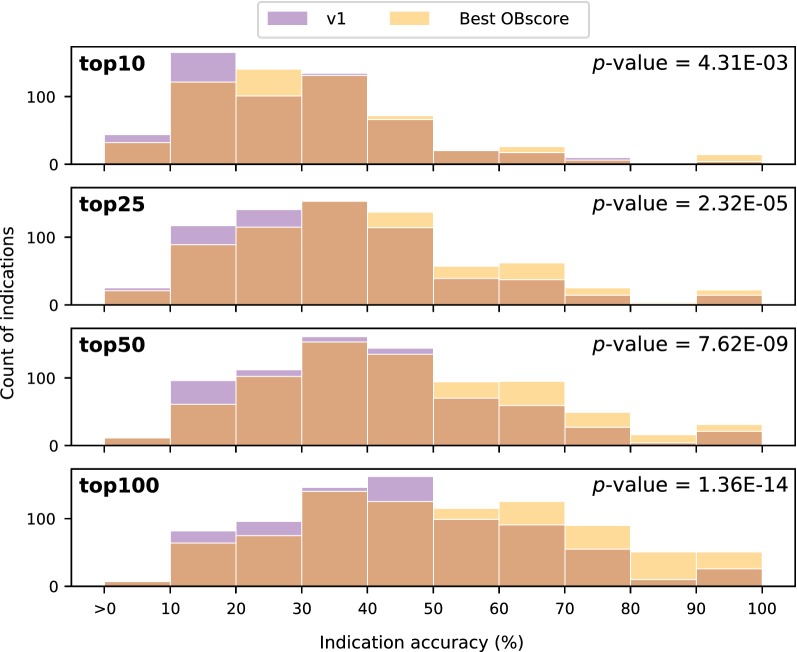
Comparison of indication accuracy distributions. A histogram of the non-zero accuracy values for the v1 (blue) and Best OBscore (yellow) pipelines at four cutoffs is plotted. The Kolmogorov–Smirnoff test, used to determine similarity (or lack thereof) of two distributions, indicates that the two pipelines have significantly different distributions of indication accuracies (p-value< 0.05). The newer v1.5 Best OB pipeline outperforms its predecessor, yielding a greater number of indications with accuracies > 50%

We calculated the Kolmogorov–Smirnoff test p-value to determine that the distribution of indication accuracies was significantly different between v1 and Best OB pipelines for all cutoffs (Fig. 2). Furthermore, the distributions in Fig. 2 show that the accuracies for Best OB, relative to v1, are skewed to the right, i.e., Best OB has a greater number of indications with accuracies > 50%.

#### Comparison of all pipelines

**Fig. 3:**
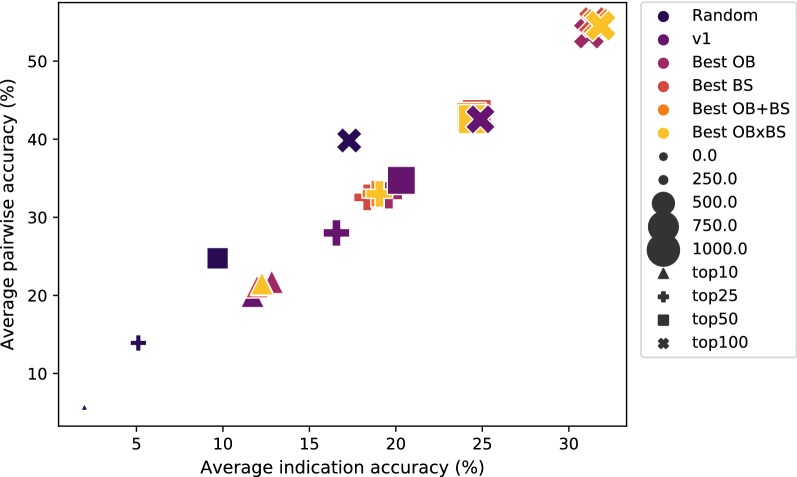
Accuracies and coverages of five CANDO pipelines at various cutoffs using different compound-protein interaction scoring metrics. Random (navy) is a random control that is calculated using the average of 100 randomly generated interaction matrices. v1 (purple) is the pipeline from the first version of the CANDO platform which used the BScore to quantify compound–protein interactions, with the chemical similarity comparison done using OpenEye ROCS [7, 16]. The Best OB pipeline (magenta) uses the highest OBscore for each compound-protein interaction. Best BS (red) uses the OBscore corresponding to the fingerprint comparison between each compound and highest BSscore binding site ligand for each protein. Best OB+BS (orange) is the highest summation of OBscore and BSscore for each compound-protein interaction, and Best OBxBS (yellow) is the highest product. The average indication and pairwise accuracies for the top10 (triangle), top25 (cross), top50 (square), and top100 (X) cutoffs are shown for each pipeline. The size of the cutoff symbol corresponds to the indication coverage. By varying the interaction scoring scheme in v1.5, we are able to discern that the Best OB protocol results in the highest benchmarking performance, particularly at the top10 and top 25 cutoffs

Figure 3 shows the accuracies and coverages of all five pipelines at different cutoffs. All scoring metrics in v1.5 did comparably well to one another and better than the pipeline used in the CANDO v1 platform. Best OB produces the highest average indication accuracy of 12.8% and 19.6% for the top10 and top25 cutoffs. At higher cutoffs, the Best BS, OB+BS, and OBxBS pipelines perform better for the average and pairwise indication accuracy metrics, with OBxBS having the highest average indication accuracy of 31.8% at the top100 cutoff.

The Best OB pipeline average indication accuracy is five times greater at the top10 cutoff when compared to the uniform distribution random control (2.0 to 12.8%). This trend remains consistent as the cutoff increases, with relative differences between random control and Best OB of 14.5, 14.7, and 13.9% for the top25, top50, and top100 cutoffs. The average pairwise accuracies and indication coverage are also higher for the Best OB pipeline when compared to the random control with a pairwise accuracy increase from 5.7 to 21.7% and coverage increase from 238 to 563 at the top10 cutoff. The relative increases between the Best OB and random control are 18.9, 17.8, and 13.7% for average pairwise accuracy and 305, 245, and 207 for the indication coverage at the remaining three cutoffs. The second random control used in this study based on a hypergeometric distribution converges to similar values as the first one.

### Discussion

Our results suggest that for preclinical validations of 25 or fewer compounds, the Best OB pipeline, which has the highest average indication accuracy, pairwise accuracy, and indication coverage at the top10 and top25 cutoffs, should be used to generate putative drug candidates. In contrast, the results show that at higher cutoffs (top50 and top100) the Best OBxBS and Best OB+BS pipelines yield better benchmarking performance, indicating that these two pipelines should be utilized for validation studies consisting of 50 or more putative drug candidates.

### Conclusions

Overall, our results illustrate the improved benchmarking performance of the updated CANDO v1.5 platform and its structure-based pipelines relative to v1, which in turn translates to greater predictive power for shotgun drug repurposing and mechanistic understanding. The top putative drug candidates and targets generated by these newer pipelines in v1.5 will aid us in discovering novel treatments and mechanisms for specific indications in future validation studies.

## Limitations

The CANDO platform is used to generate top ranking putative drug candidates for every indication. These candidates need to be experimentally validated to ensure they represent potential leads and eventually repurposed drugs for a specific indication.

Other possible scoring protocols need to be explored to determine if OBscore and BSscore most accurately quantify the compound-protein interactions. Further studies with different cheminformatics and bioinformatic tools may also provide further insight into the behaviour of the platform and are currently underway, which demonstrate that continued development of CANDO by adding novel features and pipelines greatly increases its predictive power for future drug repurposing efforts particularly when these other pipelines and optimization techniques are used in combination [11, 19].

## Additional file

**Additional file 1.** This file contains a detailed description of methods and additional results.

## Data Availability

The datasets supporting the conclusions of this article are available in the CANDO repository, http://protinfo.org/cando/results/v1_5.

## Abbreviations

CANDO: Computational Analysis of Novel Drug Opportunities
OBscore: Open babel score
BSscore: Binding site score
PDB: Protein Data Bank
RMSD: Root mean squared deviation
