# Supplementary material for: Exploration of interaction scoring criteria in the CANDO platform
Source: BMC Res Notes. 2019 Jun 7;12:318. doi: 10.1186/s13104-019-4356-3 (PMC6555930; doi:10.1186/s13104-019-4356-3)
Supplement: Supplementary file 1 — Additional file 1. This file contains a detailed description of methods and additional results. [file 13104_2019_4356_MOESM1_ESM.pdf]

SUPPLEMENTARY MATERIAL FOR RESEARCH NOTE

Exploration of interaction scoring criteria in the CANDO platform

Zackary Falls, William Mangione, James Schuler and Ram Samudrala*

*Correspondence:
ram@compbio.org
Department of Biomedical Informatics, Jacobs School of Medicine and Biomedical Sciences, University at Buffalo, 77 Goodell St., Suite 540, 14203 Buffalo, NY, US,
Full list of author information is available at the end of the article

Detailed description of methods

Putative drug library creation and indication mapping

A virtual library of FDA approved and other human use compounds was created using multiple sources including DrugBank, [1] NCGC Pharmaceutical Collection (NPC), [2] Wikipedia, and PubChem. [3] Each molecule is converted to a 3D structure using MarvinBeans molconverter v5.11.3 from ChemAxon. [4] Cactvs Chemoinformatics Toolkit [5, 6] from Xemistry was used to generate unique InChIKeys (International Chemical Identifier Keys) from the clean 3D chemical structures and each compound InChIKey was compared to all others to eliminate redundancy in the drug library. The final library contains 3,733 human use compounds, including 1387 approved drugs that map to 2,030 diseases/indications. Drug indication associations were obtained from the Comparative Toxicogenomics database. [7] 1,439 indications (out of the 2,030) are associated with two or more drugs and are used to perform the leave-one-out benchmarking of the platform described below.

Protein structure library creation and binding site predictions

A total of 46,784 solved and modeled protein structures comprise the library of potential macromolecular targets in the CANDO platform. The solved protein structures (31,145) are obtained from the Protein Data Bank (PDB) [8] and the modeled structures (15,639) are generated using the I-TASSER software [9]. The bioinformatic tool COFACTOR [10, 11, 12] is used to assess binding site similarity (Figure 1a) by comparing the protein of interest to a library of template proteins from the PDB to determine the best match for potential binding sites. [13] The metric that defines the local structure and sequence similarity between the target and template binding sites is the binding site similarity score (BSscore). [12]

Chemical fingerprint identification and comparison

The FP4 fingerprinting method in Open Babel is used to determine the cheminformatic structural similarity score (OBscore) [14] between all binding site ligands from COFACTOR and structures in the putative drug library (Figure 1b). We chose Open Babel to determine structural fingerprints in CANDO v1.5 [15] as it is an established suite of open source software. [14]

Generation of compound-proteome interaction signatures

We quantify the compound-protein interaction strength using combinations of the OBscore and/or BSscore described above. When applied to the corresponding libraries, this generates a compound-protein interaction matrix (Figure 1c), where

each row of this matrix, *i.e.* the compound-proteome interaction signature, describes how each compound interacts with the entire multiorganism protein library.

In CANDO v1.5, we use the OBscore and BScore to populate the interaction matrix for the following pipelines: Best OB, Best BS, Best OB+BS, and Best OBxBS. The values in the matrix for each compound-protein interaction in the first two pipelines use the OBscore; Best OB is the highest OBscore between the compound and all predicted binding site ligands for each protein, while Best BS is the OBscore that corresponds to the best local binding site prediction using COFACTOR. The last two pipelines involve adding and multiplying the OBscore and BSscore for each compound-protein interaction; the highest sum or product between the compound and the predicted binding site ligands was chosen as the interaction score.

Calculating interaction signature similarities

The similarity between every compound-proteome interaction signature is compared to all other signatures (Figure 1d) using the root-mean-square deviation (RMSD). This procedure generates a symmetric matrix (with zeroes along the diagonal) of similarity scores that are hypothesised to represent how functionally similar each compound is to all the others in the context of the protein structure library.

Ranking drug lists and benchmarking metrics

The RMSDs in each row of the compound-compound similarity matrix are sorted to yield ranked similarity lists for each compound (Figure 1e). Each drug associated with an indication is left/held out and checked to see if it is captured within a certain cutoff in the ranked list to any of the other remaining ones [associated with that indication] (Figure 1f). The cutoffs used typically are top10, top25, top50, and top100, reflecting the top ranked 10-100 similar compounds for a given drug.

This procedure is repeated iteratively for all drugs associated with every indication for a particular cutoff, resulting in the indication accuracy. Mathematically, indication accuracy is calculated using the formula $\frac{c}{d} \cdot 100$, where c is the number of times at least one drug with the same indication was captured within a particular cutoff and d is the total number of drugs approved for that indication. Taking the mean of these accuracies (for all 1439 indications with at least two approved drugs) gives the average indication accuracy for a pipeline at a particular cutoff.

The other benchmarking metrics used are the average pairwise accuracy which is a weighted average of all indication accuracies based upon the number of approved drugs for each indication, and indication coverage, which is the number of indications with a non-zero accuracy (i.e., at least one approved drug that was left out was successfully recaptured within a cutoff).

Generation of random controls

We devised two types of random controls. First, we generated random compound-proteome interaction matrices to compare the efficacy of v1 and the v1.5 pipelines against a control. For each compound-protein interaction score we randomly selected a value from a uniform distribution between 0.0 and 1.0 to populate a 3,733 (compounds) by 46,784 (proteins) interaction matrix. We benchmarked this matrix, as discussed in a previous section, to ascertain the benchmarking metrics (average

indication accuracy, average pairwise accuracy, and indication coverage) for all cutoffs (top10, top25, top50, and top100). This protocol was repeated 100 times and the resulting averaged metrics were used as the random control.

As a second random control, we calculated the hypergeometric distribution for the leave-one-out benchmarking protocol at each cutoff using Equation 1.

$$p = 1 - \frac{\binom{K}{k}\binom{N-K}{n-k}}{\binom{N}{n}} \tag{1}$$

where p is the probability of recapturing at least one drug approved (k) for the same indication as the "left out" drug in the top$n$ cutoff, considering a population (N) of 3732 compounds and K being the number of approved drugs for the indication. The probability was calculated and averaged across all indications, for which the number of approved drugs, K, varies.

Additional results

Increasing the signal in the matrices and pipeline in CANDO v1.5

Using the v1.5 pipeline we decreased the noise in the compound-proteome interaction matrix relative to v1. Specifically, in v1 more than 800 compounds in our library had completely null interaction signatures (as shown in Figure S1), i.e., every compound-protein interaction for the $\approx$ 800 compounds received a score of 0.0. We reduced that number to less than 50 compounds, all of which are chemical ions that fail in the current version of the bioinformatic docking protocol. In addition, $\approx$ 66% of all compound-protein interaction scores in v1 was assigned a score of 0: Each compound-proteome interaction signature in v1, on average, contained 38,492/46,784 (median = 36,778) null or zero interaction scores, whereas the average number of null interaction scores within v1.5 has decreased significantly to 792 (median = 40).

These changes resulted in an increase from 11.7% in v1 to 12.8% in v1.5 for the top10 average indication accuracy, corresponding to 9.4% increase between versions. Furthermore, a greater improvement in accuracy between versions was observed at higher cutoffs, with a 25% increase for the top100 average indication accuracy. This indicates a greater capability of our platform to recapture known drugs for each indication, as well as to more accurately predict putative repurposable drugs for all indications.

Variation of OBscore and BSscore threshold values

In v1, we used a threshold value of 1.1 for the ROCSscore and BSscore to determine if a protein-compound interaction would occur, based on an analysis of structure-ligand complexes. [16] For v1.5, we benchmarked the Best OB matrix for each incremental increase in OBscore (0 to 1) and BSscore (0 to 2) values individually to determine how these thresholds influence the overall benchmarking result.

As shown in Figure S2, both OBscore and BSscore thresholds were increased incrementally and independently from 0 to their corresponding theoretical maxima of 1 and 2 respectively. As the OBscore threshold is increased, the resulting accuracies and coverages decrease, eventually approaching zero (Fig. S2). This is because the

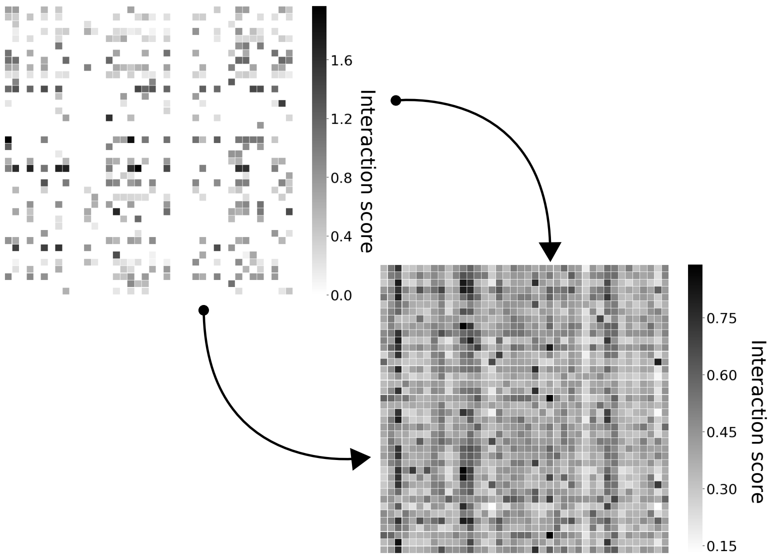

Additional file 1: Figure S1 Increased signal in v1.5 interaction matrix. In v1, about 800 compounds had all-zero signatures, wheres in v1.5 all of these compounds have non-zero signatures. The average indication accuracy increased 1.1% (from 11.7% to 12.8%) for the top10 cutoff, and by 6.3% for the top100 cutoff (from 24.9% to 31.2%). Upgrading the CANDO v1 pipeline to v1.5 has resulted in the signal-to-noise ratio being increased (i.e., more interactions calculated) by about 20% for the compound-proteome interaction matrix.

ligand and compound must have near identical chemical similarity to have a high OBscore. However, few of the compound in the CANDO library are chemically similar to the ligands present in the binding sites of the template proteins from COFACTOR, let alone identical (where the OBscore would be 1). The results from Figure S3 indicates that as the OBscore threshold increases, the number of non-zero OBscores in the interaction matrix will decrease, and the average indication accuracies and coverage will decrease correspondingly (Figure S2).

In contrast, the incremental increase in the BSscore threshold does not significantly affect benchmarking performance (Fig. S2). A large portion of the protein structure library used by the CANDO platform consists of solved PDB structures that overlap highly with the COFACTOR template library. This results in a considerable amount of signal remaining at the highest thresholds (Figure S3). However, the indication coverage appears to decrease by ≈ 30 as the threshold is increased, meaning the high OBscore cutoff is resulting in the average indication accuracies to remain consistent, however, the accuracy is averaged over fewer number of indications.

References

1. Knox, C., Law, V., Jewison, T., Liu, P., Ly, S., Frolkis, A., Pon, A., Banco, K., Mak, C., Neveu, V., Djoumbou, Y., Eisner, R., Guo, A.C., Wishart, D.S.: Drugbank 3.0: a comprehensive resource for 'omics' research on drugs. Nucleic Acids Research **39**(suppl_1), 1035–1041 (2011). doi:10.1093/nar/gkq1126
2. Huang, R., Southall, N., Wang, Y., Yasgar, A., Shinn, P., Jadhav, A., Nguyen, D.-T., Austin, C.P.: The ncgc pharmaceutical collection: A comprehensive resource of clinically approved drugs enabling repurposing and chemical genomics. Science Translational Medicine **3**(80), 80–168016 (2011). doi:10.1126/scitranslmed.3001862
3. Li, Q., Cheng, T., Wang, Y., Bryant, S.H.: Pubchem as a public resource for drug discovery. Drug Discovery Today **15**(23), 1052–1057 (2010). doi:10.1016/j.drudis.2010.10.003

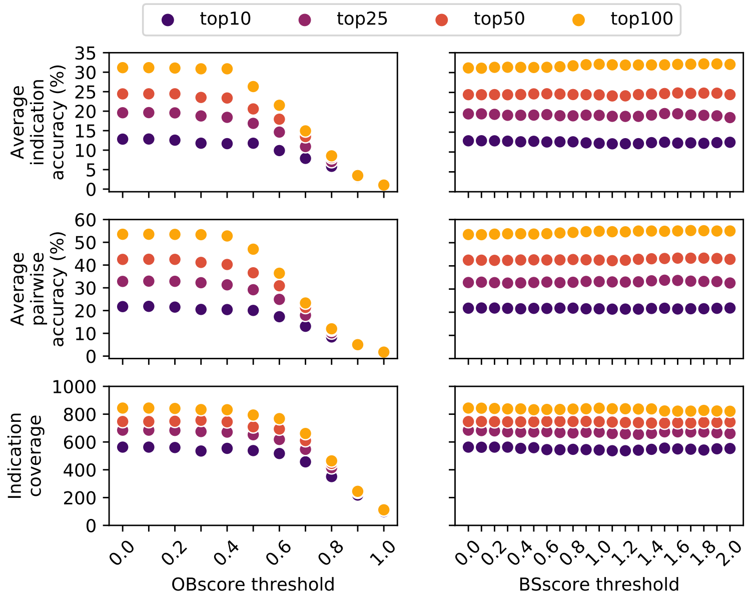

Additional file 1: Figure S2 Change in benchmarking performance when varying the OBscore or BSscore thresholds. The OBscore and BSscore thresholds were set to a value between 0.0-1.0 and 0.0-2.0, respectively; scores above the threshold value were used to populate the compound-proteome interaction matrix and scores below were set to zero. Matrix generation and benchmarking was performed over the full range at increments of 0.1 for OBscore and 0.2 for BSscore. The average indication accuracy, average pairwise accuracy, and indication coverage are shown for four compound-compound similarity list cutoffs: top10 (purple), top25 (magenta), top50 (red), and top100 (yellow). The plots for the OBscore show that as the threshold is increased towards the theoretical maximum of 1.0, the resulting accuracies diminish to nearly 0%. This is because as the threshold becomes more stringent, the number of compounds that have a strong similarity (OBscore greater than the threshold) to the binding site ligands approaches zero, therefore more of the drugs will have near–zero proteome signatures. If there is no signal to discern compound–proteome interaction signature similarity, then the benchmarking produces decreasing accuracies with each increment to the threshold value. In contrast, the BSscore plots show that as the threshold is increased toward the theoretical maximum of 2.0, there is negligible fluctuation in the average indication and pairwise accuracies because there exist predicted binding sites for most proteins in our library that receive a BSscore of 2.0 from COFACTOR. Based upon these results, we used a lower cutoff for the OB and BSscores to obtain the best benchmarking performance.

4. Garcia-Cordoba, F., Garcia-Santos, J., González, G.D., Garcia-Geronimo, A., Muñoz, F.Z., Peñalver, F.H., Del, L.B.A.: Decrease of unnecessary chest x-rays in intensive care unit: application of a combined cycle of quality improvement. Medicina intensiva **32**(2), 71–77 (2008)
5. Ihlenfeldt, W.D., Takahashi, Y., Abe, H., Sasaki, S.-i.: Computation and management of chemical properties in cactvs: An extensible networked approach toward modularity and compatibility. Journal of chemical information and computer sciences **34**(1), 109–116 (1994)
6. Xemistry chemoinformatics (2012). http://www.xemistry.com
7. Davis, A.P., Murphy, C.G., Johnson, R., Lay, J.M., Lennon-Hopkins, K., Saraceni-Richards, C., Sciaky, D., King, B.L., Rosenstein, M.C., Wiegers, T.C., *et al.*: The comparative toxicogenomics database: update 2013. Nucleic acids research **41**(D1), 1104–1114 (2012)
8. Berman, H.M., Westbrook, J., Feng, Z., Gilliland, G., Bhat, T.N., Weissig, H., Shindyalov, I.N., Bourne, P.E.: The protein data bank. Nucleic Acids Research **28**, 235–242 (2000)
9. Yang, J., Yan, R., Roy, A., Xu, D., Poisson, J., Zhang, Y.: The i-tasser suite: protein structure and function prediction. Nature methods **12**(1), 7 (2015). doi:10.1038/nmeth.3213
10. Zhang, C., Freddolino, P.L., Zhang, Y.: Cofactor: improved protein function prediction by combining structure, sequence and protein–protein interaction information. Nucleic Acids Research **45**(W1), 291–299 (2017). doi:10.1093/nar/gkx366
11. Roy, A., Yang, J., Zhang, Y.: Cofactor: an accurate comparative algorithm for structure–based protein function annotation. Nucleic Acids Research **40**(W1), 471–477 (2012). doi:10.1093/nar/gks372
12. Roy, A., Zhang, Y.: Recognizing protein–ligand binding sites by global structural alignment and local geometry refinement. Structure **20**(6), 987–997 (2012). doi:10.1016/j.str.2012.03.009
13. Yang, J., Roy, A., Zhang, Y.: Biolip: a semi-manually curated database for biologically relevant ligand–protein interactions. Nucleic acids research **41**(D1), 1096–1103 (2012)

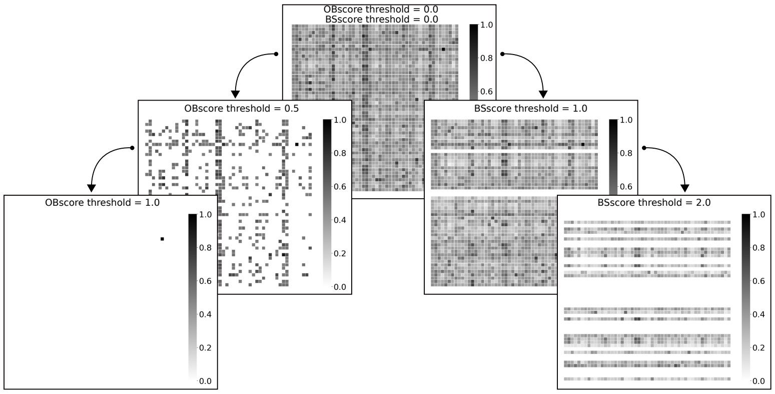

Additional file 1: Figure S3 Increase in signal in the interaction matrices as a function of score thresholds. A small section of the matrix that corresponds to no threshold for either BSscore or OBscore is shown top centre. A two step threshold increase of OBscore ($0.0 \rightarrow 0.5 \rightarrow 1.0$) is shown to its left and a two step threshold increase of the BSscore ($0.0 \rightarrow 1.0 \rightarrow 2.0$) is shown to its right. As the OBscore threshold is increased, the resulting matrix contains many null values (interaction score of zero) leading to diminished accuracies (Figure S2. In contrast, increasing the BSscore threshold yields a sufficient amount of signal, with the corresponding benchmarking performance largely unchanged. Overall, these results indicate that using lower BSscore and OBscore thresholds yield better benchmarking performance.

14. O'Boyle, N.M., Banck, M., James, C.A., Morley, C., Vandermeersch, T., Hutchison, G.R.: Open babel: an open chemical toolbox. Journal of Cheminformatics **3**(1), 33 (2011). doi:10.1186/1758-2946-3-33
15. Hawkins, P.C., Skillman, A.G., Nicholls, A.: Comparison of shape-matching and docking as virtual screening tools. Journal of medicinal chemistry **50**(1), 74–82 (2007)
16. Sethi, G., Chopra, G., Samudrala, R.: Multiscale modelling of relationships between protein classes and drug behavior across all diseases using the cando platform. Mini reviews in medicinal chemistry **15**(8), 705–717 (2015)
